# Perfusion in Organ-on-Chip Models and Its Applicability to the Replication of Spermatogenesis In Vitro

**DOI:** 10.3390/ijms23105402

**Published:** 2022-05-12

**Authors:** Sholom Shuchat, Gilad Yossifon, Mahmoud Huleihel

**Affiliations:** 1 Faculty of Mechanical Engineering, Technion–Israel Institute of Technology, Haifa 3200003, Israel; sholomber@gmail.com (S.S.); gilad.yossifon@gmail.com (G.Y.); 2 School of Mechanical Engineering, University of Tel Aviv, Tel Aviv 6997801, Israel; 3 The Shraga Segal Department of Microbiology, Immunology and Genetics, Faculty of Health Science, Ben-Gurion University of the Negev, Beer Sheva 8410501, Israel; 4 The Center of Advanced Research and Education in Reproduction (CARER), Ben-Gurion University of the Negev, Beer Sheva 8410501, Israel; 5 Faculty of Health Sciences, Ben-Gurion University of the Negev, Beer Sheva 8410501, Israel

**Keywords:** microfluidics, organ/organoid-on-a-chip, microfluidics, perfusion, male-fertility, spermatogenesis, sperm, post-cancer fertility

## Abstract

Organ/organoid-on-a-chip (OoC) technologies aim to replicate aspects of the in vivo environment in vitro, at the scale of microns. Mimicking the spatial in vivo structure is important and can provide a deeper understanding of the cell–cell interactions and the mechanisms that lead to normal/abnormal function of a given organ. It is also important for disease models and drug/toxin testing. Incorporating active fluid flow in chip models enables many more possibilities. Active flow can provide physical cues, improve intercellular communication, and allow for the dynamic control of the environment, by enabling the efficient introduction of biological factors, drugs, or toxins. All of this is in addition to the fundamental role of flow in supplying nutrition and removing waste metabolites. This review presents an overview of the different types of fluid flow and how they are incorporated in various OoC models. The review then describes various methods and techniques of incorporating perfusion networks into OoC models, including self-assembly, bioprinting techniques, and utilizing sacrificial gels. The second part of the review focuses on the replication of spermatogenesis in vitro; the complex process whereby spermatogonial stem cells differentiate into mature sperm. A general overview is given of the various approaches that have been used. The few studies that incorporated microfluidics or vasculature are also described. Finally, a future perspective is given on elements from perfusion-based models that are currently used in models of other organs and can be applied to the field of in vitro spermatogenesis. For example, adopting tubular blood vessel models to mimic the morphology of the seminiferous tubules and incorporating vasculature in testis-on-a-chip models. Improving these models would improve our understanding of the process of spermatogenesis. It may also potentially provide novel therapeutic strategies for pre-pubertal cancer patients who need aggressive chemotherapy that can render them sterile, as well asfor a subset of non-obstructive azoospermic patients with maturation arrest, whose testes do not produce sperm but still contain some of the progenitor cells.

## 1. Introduction

In the last two decades, organ-on-a-chip (OoC) technology has become an increasingly powerful tool for the study of various organs in the body. The underlying premise is to create an in vitro environment at the scale of microns that mimics the function or structure of an organ [1]. Generally, they are based on microfluidic technologies [1]. The ability to design structures on a cellular scale provides the ability to engineer in vitro a replication of the in vivo environment. There are various levels of complexity, ranging from models with a single cell type on a membrane, to complex multi-cellular bioprinted structures. Some key advantages of the small scales are the ability to replicate the complex architecture of the organs, to accurately control the microenvironment [2], such as local chemical gradients, and to supply nutrition to dense cellular structures, while removing waste [2], thus reducing atrophy and necrosis [3]. Mechanical and electrical stimuli can also be introduced to mimic the in vivo environment and study their physiological effects (see Griffith et al. [4], Kaarj [5], Chen et al. [6], and Moraes et al. [7] for in depth reviews). For a more in-depth overview of OoC technology, see the following reviews by Tian et al. [1], Zhang et al. [2], and Samad et al. [8].

Incorporating active fluid flow (perfusion) in a microfluidic chip, as opposed to static culture conditions, opens a wide range of possibilities that can improve the culture results and provide the ability to mimic organ functions that involve flow [9]. Fluid flow can be used to improve the nutrition supply and cellular crosstalk. It can also be used to provide physical stimuli to the cells [4]. This review examines how active flow is incorporated into various OoC models. It then summarizes a number of relevant approaches that have been used in the study of in vitro spermatogenesis. Lastly, it suggests possible implementations of OoC methods incorporating active flow, to improve modeling of the testis in vitro.

## 2. Types of Microfluidic OoC Setups

**Figure 1:**
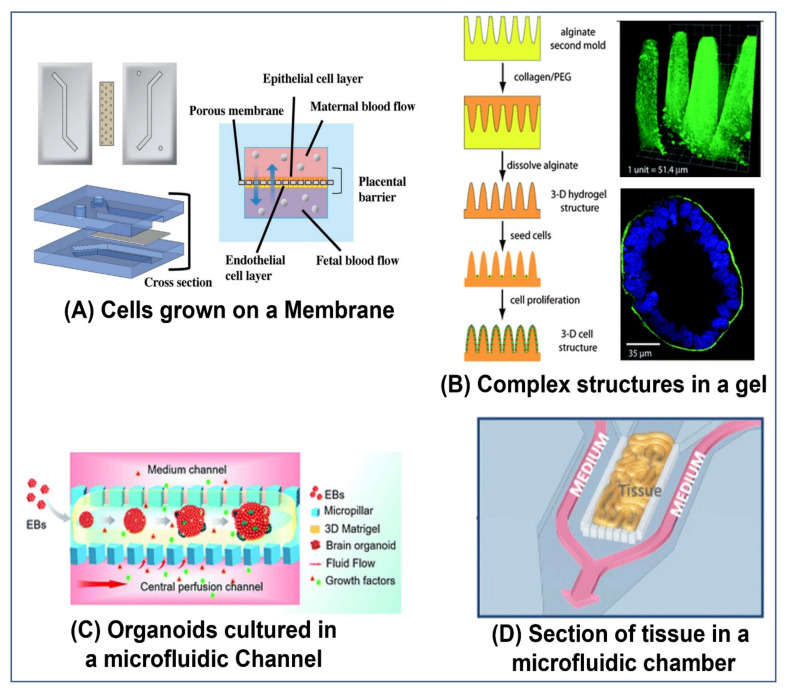
**Types of organ-on-a-chip models:** (**A**) A model that cultures cells on a porous membrane that divides the microchannel. Specifically, the image is of a placenta-on-a-chip model to model the placenta barrier, which interfaces between the maternal epithelium and the fetal endothelium. The channel is separated into two channels by a porous membrane. On one side, human umbilical vein endothelial cells (HUVECs) are cultured, representing the fetal endothelium. Maternal epithelium trophoblasts cells (BeWo) were cultured on the second side of the membrane, representing the maternal epithelium [11]. Reproduced with permission from Global Challenges; published by Wiley, 2019, under a creative commons CC BY license (https://creativecommons.org/licenses/ accessed on 1 May 2022). (**B**) Cells from the colon carcinoma cell line Caco-2 were seeded on collagen gel that was molded to achieve the morphology of intestinal villi. The cells were cultured for three weeks and formed a confluent layer, thus mimicking the intestinal epithelium [12]. Reproduced with permission from Lab Chip; published by MDPI, 2011. (**C**) Brain organoids derived from human induced pluripotent stem cells (hiPSC) were cultured in a microfluidic chip with perfusion flow in the side channels. The expression of the cortical layer markers (TBR1 and CTIP2) was higher in the perfused chips than in the static controls, indicating that the active fluid flow promoted brain organogenesis [13]. Reproduced with permission from RSC Advances; published by RSC, 2019, under a Creative Commons BY NC 3.0 license (https://creativecommons.org/licenses/by-nc/3.0 accessed on 1 May 2022). (**D**) Testicular tissue fragments were cultured in the center chamber of a microfluidic chip. Media was continuously perfused in the side channels. Using this setup, spermatogenesis was maintained for six months [14]. Reproduced with permission from Biochemical and Biophysical Research Communications; published by Elsevier, 2018.

There are a number of different ways OoCs are setup. Some simply use the chip as a micro-sized petri-dish in which to culture the cells, tissue segments, or organoids (as in Figure 1C,D). Other methods use the chip itself or substructures in the gels to provide a structure and define the organoid or organ model (as in Figure 1A,B). Cells for such models are often from animals or immortalized human cell-lines. Thus, OoC models can reduce the need for live animal models. However, OoC technology can also be used as part of personalized medicine and treatments, as in the glioblastoma-on-a-chip model developed by Yi et al. [10]. Similarly, OoC models of the testis- could possibly be used in the future, to culture spermatogonial precursor cells from human patients and develop them into mature fertile sperm.

In some methods, a gel or liquid containing the cells is injected into the chip, where it proceeds to self-assemble and form an organoid or other complex structure. The organoid may also be formed off-chip, for example in a well plate, and then transferred to the chip, as done by Wang et al. [13] (see Figure 1C), Baert et al. [15], and Nashimoto et al. [16]. An advantage of forming the organoid off the chip is the ability to consistently produce organoids of the same size and composition, which is important for repeatability and optimization. However, there are also methods, to do this on-chip, as demonstrated by Lee et al. [17] and our design in Figure 8C. The shape, size, and other characteristics of the chip can influence the final form of the organoid [18]. Additionally, external stimuli, whether mechanical, electrical, or chemical, can also be used to guide the development and organization of the cells [18]. For example, in a chip designed to promote the formation of vasculature, location-specific gradients of factors, such as vascular endothelial growth factor (VEGF), can be employed [19]. Similar chips are also used to culture sections of tissue for organ culture, as demonstrated by Yamanaka et al. [14] (See Figure 1D) and Liu et al. [20].

Other methods use the structure of the chip as a platform for growing the cells. For example, in the simulation of blood-tissue barriers, the cell types found within an organ could be grown on one side of a porous membrane, while the external epithelial cells are grown on the other side of the membrane, such as the placental barrier replicated by Pemathilaka et al. [11] (see Figure 1A), and the intestinal wall replicated by Jalili-Firoozinezhad et al. [21] (see Figure 5A). This effectively creates two chambers that simulate the environment within and without the organ.

**Figure 2:**
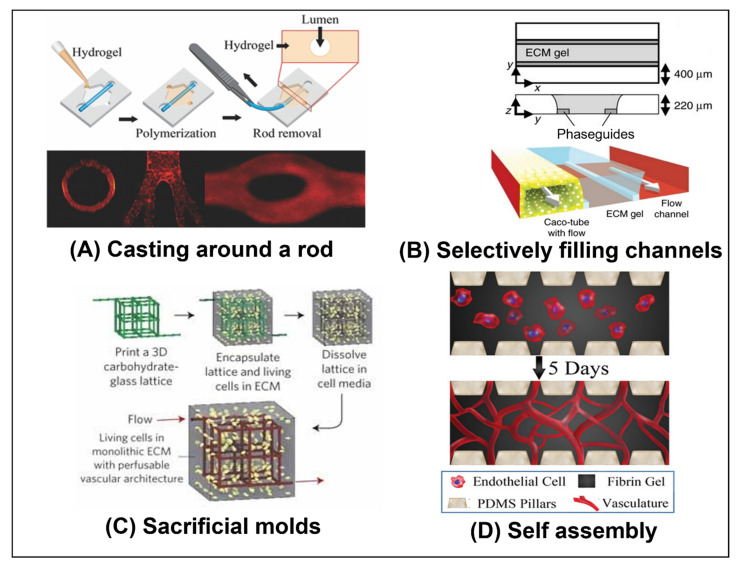
**Methods to create tubular structures in organ-on-a-chip models:** (**A**) A gel in a liquid state is cast around a flexible PDMS rod. Once the gel solidifies, the rod is removed, leaving a tubular hollow in the gel. As an example, the resulting channels were lined with human umbilical vein endothelial cells (HUVEC) [27]. Reproduced with permission from Advanced Healthcare Materials; published by Wiley, 2018. (**B**) Phaseguides (a short protrusion spanning the length of the channel) are used to guide a gel in liquid form to fill only the center of the channel. Once solidified, it effectively divides the channel into two. In this experiment, cells from the human colon adenocarcinoma cell line Caco-2 were seeded into one channel, where they adhered to the walls. When confluence was achieved, the permeability of the barrier was tested [24]. Reproduced with permission from Nature Communications; published by Springer Nature, 2017, under a Creative Commons BY 4.0 license (http://creativecommons.org/licenses/by/4.0/ accessed on 1 May 2022). (**C**) A carbohydrate-glass filament (made up of a mixture of sugars) was printed in the shape of the desired vascular network. The printed network was then encapsulated in a gel containing cells. Once the gel was set, the carbohydrate was dissolved in culture medium, and the channels could then be perfused [28]. Reproduced with permission from Nature Materials; published by Springer Nature, 2012. (**D**) A microfluidic culture chip was filled with a fibrin gel containing HUVECs and normal human lung fibroblast (NHLF) cells. After five days of culture, the cells formed a fully developed and perfusable vascular network [29]. Reproduced with permission from Advanced Materials Technologies; published by Wiley, 2019.

**Figure 3:**
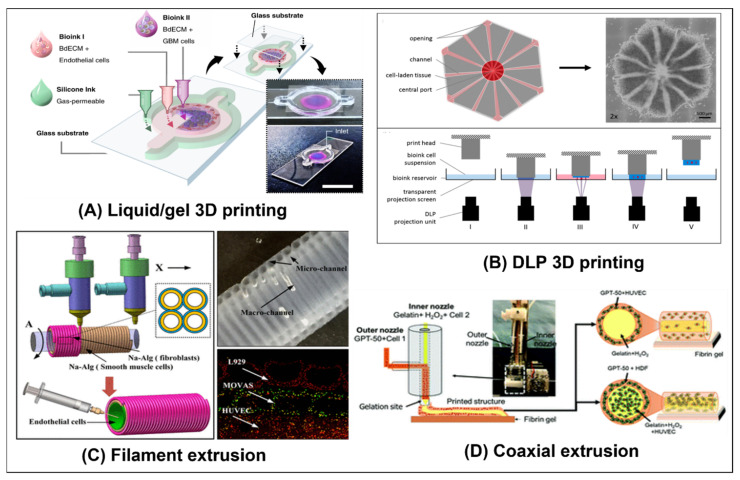
**Methods of bioprinting:** (**A**) Liquid bio-inks impregnated with different types of cells were 3D printed onto a glass slide, to create a patient specific glioblastoma-on-a-chip model. Patient derived cancer cells were printed in the chip’s center, surrounded by a layer of vascular cells. The chips were then subjected to various cancer treatments and the differences in their response reflected the differences in the clinical responses of the patients to cancer treatments [10]. Reproduced with permission from Nature Biomedical Engineering; published by Springer Nature, 2019. (**B**) Liver organoids that mimic the sinusoidal structure of liver lobules were printed using a DLP 3D printer. The lobules contained a perfusable network [39]. Reprinted with permission from Genes; published by MDPI, 2019. (**C**) Micro-tubes made from cell-laden alginate gels were extruded, using a coaxial nozzle, onto a rotating glass rod to create a macro-vessel. The lumen of the macro vessel was then coated with collagen. Finally, HUVECs were introduced into the macro channel. The result is a vessel with multiple layers of different cell types (fibroblasts, smooth muscle cells, and endothelial cells). The outer micro-channels could also be perfused [42]. Reprinted with permission from 3D Bioprinting of Vessel-like Structures with Multilevel Fluidic Channels, ACS Biomater. Sci. Eng., vol. 3, no. 3, pp. 399–408. Copyright 2017, American Chemical Society. (**D**) A vascular vessel was extruded onto a fibrin gel biopaper using a coaxial nozzle and bio-inks containing cells. Vessels could be printed with HUVECs embedded in the walls and a sacrificial core to create a perfusable lumen. Alternatively, vessels could be printed with human dermal fibroblasts (HDFs) embedded in the walls and a core containing gelatin laden with HUVECs [37]. Reproduced with permission from Biomaterials Science; published by RSC, 2019.

**Figure 4:**
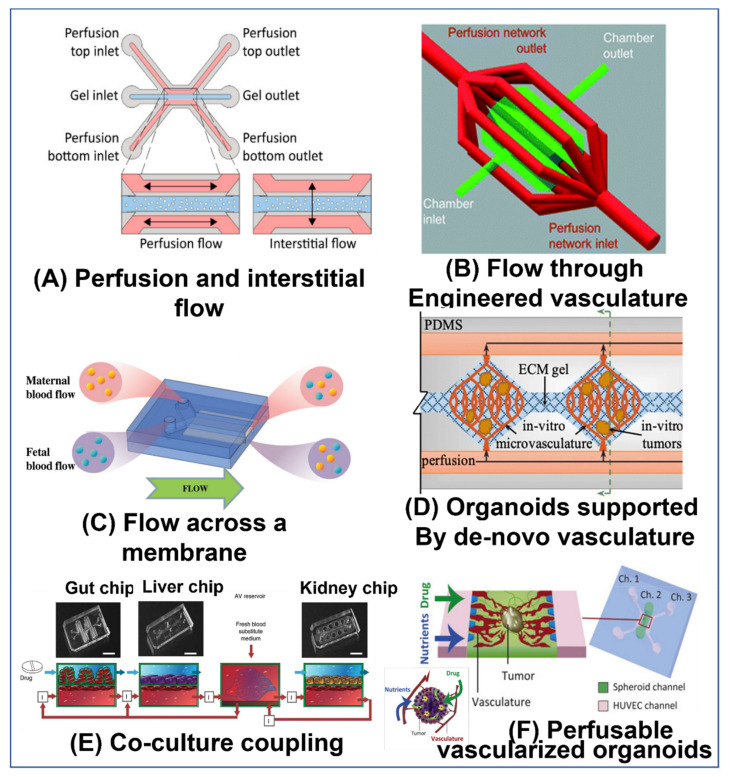
**Organ-on-a-chip models that incorporate perfusion:** (**A**) Pancreatic ductal adenocarcinoma cells (PDAC) were cultured in a microfluidic chip. Perfusion through the side media channels was controlled by a programmable rocker. To mimic interstitial flow, perfusion across the cell culture chamber was induced by filling the media reservoirs to different heights. The interstitial flow was used to mimic the intratumoral fluid pressure present in such tumors in vivo [51]. Reprinted with permission from International Journal of Molecular Science; published by MDPI, 2019. (**B**) A culture chip with an engineered perfusion network was 3D printed using an SLA printer. HT-29 cells (a human colorectal adenocarcinoma cell line with an epithelial morphology) were seeded in the chamber, and media was continuously perfused through the perfusion network. The perfused cultures had a higher percentage of live cells after one week of culture compared to static conditions [22]. Reproduced with permission from Lab on a Chip; published by RSC, 2017. (**C**) A membrane based OoC model of the placenta barrier (as described above in Figure 1A) was continuously perfused with media. Caffeine was then added to the flow on the maternal side, and the resulting concentration on each side was measured until reaching a steady state at around seven hours [11]. Reproduced with permission from Global Challenges; published by Wiley, under a Creative Commons CC BY license (https://creativecommons.org/licenses/ accessed on 1 May 2022). (**D**) Human endothelial colony forming cell-derived ECs (ECFC-ECs), NHLF cells, and cancer cells were seeded in a microfluidic device. Constant interstitial flow was maintained to induce vasculogenesis. Eventually the vessels anastomosed to the main channel, allowing the perfusion of the cell culture via the vasculature [43]. Image reproduced from [53]. Image reproduced with permission from Journal of Nanobiotechnology; published by Springer Nature, under a Creative Commons BY 4.0 license (http://creativecommons.org/licenses/by/4.0/ accessed on 1 May 2022). (**E**) A co-culture model that fluidically couples, via constant perfusion, gut, liver, and kidney OoC models. The models each have an organ model chamber on one side of a porous membrane. The other side of the membrane is lined with organ-specific endothelial cells. Culture media is continually circulated through the endothelial cell compartments coupling the models. The combined model was used to study the pharmacokinetics (PKs) and pharmacodynamics (PDs) of drugs such as nicotine [47]. Reproduced with permission from Nature Biomedical Engineering; published by Springer Nature, 2020. (**F**) NHLF, HUVEC, and cancer cells were cultured off-chip, to create vascularized tumor organoids. They were then seeded in a fibrin–collagen gel and seeded in the center channel of the chip. HUVECs were seeded in the side channels, and the chip was cultured in hypoxic conditions until vascular networks formed in the gel and anastomosed with the vasculature network in the organoids. Media could then be perfused through the organoids via these networks. The effect of the drug Paclitaxel on the organoids was tested [16]. Reproduced with permission from Biomaterials; published by Elsevier, 2020.

More advanced methods often create a sub structure within a chip. This is frequently accomplished by using a gel that can be molded and cured to form complex geometrical structures, using sacrificial gels, or a combination of the two (as demonstrated by Sung et al. [12], see Figure 1B), directly 3D printing a structure (as demonstrated by Zhang et al. [22], see Figure 4B), or by using bioprinting techniques (as demonstrated by Yi et al. [10], see Figure 3A). Cells can be seeded, both within the gel that forms the structure itself (as demonstrated by Huang et al. [23]), or on the walls of the hollow parts of the structure (as demonstrated by Trietsch et al. [24], see Figure 2B). These methods can be used to create multi-layered structures with various cell types and scaffolds for more advanced models of the organ.

In some cases, the more complex the model and the more accurately its in vivo spatial structure and cellular composition are replicated, the better it can mimic the in vivo environment. However, sometimes when the goal is to model a specific effect, it may be better to have a controlled geometry or subset of cells, to be able to accurately compare the conditions between models. For example, when studying the effect of shear stress on the walls of blood vessels, it may be better to have a controlled tubular geometry, or even a layer of cells on a flat membrane, rather than a more in -vivo-like branched vascular structure.

## 3. Methods of Creating Channels, Tubes, Lumens, and Other Structures in Gels

To facilitate flow within the culture compartments of the chips, networks of tubes and channels are often incorporated into the cell culture channels, similar to vessel-on-a-chip models [25]. This can be accomplished through a number of different means.

The simplest method is using a platform that allows only selected areas of the chip to be filled with gels. This can be accomplished by using posts or other types of capillary-action-based barriers that restrict the gel to specific areas during filling, while it is still in a liquid state. Once the chip is filled, the gel is solidified by changing the temperature, using a curing agent, ultraviolet (UV) light etc., as demonstrated by Huang et al. [23], Farahat et al. [26], and Trietsch et al. [24] (see Figure 2B).

Other methods make use of molds. In the case of removable molds, a mold of the tube is placed in the compartment, which is then filled with a gel in liquid form. When the gel has solidified, the mold is removed, leaving a tube in the gel. The mold can be rigid, such as a needle, as demonstrated by Nguyen et al. [30], and Price et al. [31], or flexible, for example polydimethylsiloxane (PDMS), as demonstrated by Jiménez-Torres et al. [27] (see Figure 2A).

To enable more complex designs, sacrificial materials are used to make the molds. A structure is formed using the sacrificial material, and a gel is cast around the structure. Once the gel is cured, the sacrificial material is removed, leaving a complex structure of hollows within the gel. The sacrificial structure can be made of materials such as alginate [32], gelatin, as used by Golden et al. [33] and Baker et al. [19], or sugar, as used by Miller et al. [28] (see Figure 2C), etc.

In the case of structures containing vasculature, vascular endothelial cells (VEC) can be encouraged to self-assemble into a vascular structure. There are various approaches to achieve such a structure (see Moses et al. [25], Zhang et al. [34], and Fritschen et al. [35] for more extensive reviews), for example, the use of angiogenic factors, such as VEGF, and mechanical flow stimulation. Supporting cells, such as fibroblasts, are also often needed [25]. By controlling the location and the gradients of these factors and cells, the vascular networks can be formed in specific locations [19].

As 3D bioprinting technology improves, it has become possible to create increasingly complex perfusable structures [36]. Various cell types that are suspended in an appropriate bio-ink can be 3D printed into an organ specific shape, with each cell type placed in the desired location. There are a number of approaches to bioprinting, including extrusion (as used by Hong et al. [37] (see Figure 3D) and Robinson et al. [38] (see Figure 7B)), stereolithography (SLA) (such as laser-based SLA used by, Zhang et al. [22] (see Figure 4B), and digital light processing (DLP) used by Grix et al. [39], see Figure 3B)), and even laser-induced forward technology (LIFT), which can print a single cell at a time [40]. Various extracellular matrix (ECM) components can be printed, and there are many different types of bio-inks and gels that can be used. Using these techniques, complex perfusable organoids containing multiple features of an organ can be printed. For a comprehensive overview of bioprinting, see Sun et al. [41], Ma et al. [36], and Yanagawa et al. [32].

For the bioprinting of tubular structures a number of methods can be used [36]. Sacrificial gels can be printed in various configurations, as demonstrated by Miller et al. [28] (see Figure 2C), or a relatively stiff and stable gel can be directly printed with a hollow tubular network, as demonstrated by Zhang et al. [22] (see Figure 4B). Multi-layered tubes can also be printed by winding filaments around a rod, as demonstrated by Gao et al. [42] (see Figure 3C). A much simpler option, however, is the use of a coaxial nozzle that can print several concentric layers of varying thickness, as demonstrated by Hong et al. [37] (see Figure 3D) and Robinson et al. [38] (see Figure 7B). A tube of any desired length can thus be extruded. The tube can then be a structure onto itself or it can be a part of a larger structure. By utilizing a sacrificial central core, the tube can remain perfusable [37,38].

**Figure 5:**
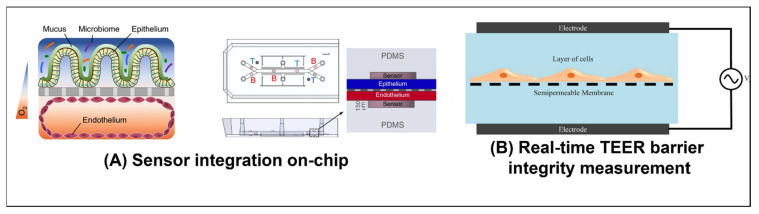
**Sensing on-chip:** (**A**) Human Caco2 intestinal epithelial cells were grown in the upper section of the channel and human intestinal microvascular endothelial cells (HIMECs) in the lower section. They each then formed a confluent layer. Gut microbia were then introduced in the gut-side of the chip. Before seeding the cells, oxygen-sensitive and optical isolating particles were mixed to make optical oxygen sensor spots on the chip. The oxygen levels could then be monitored non-invasively, throughout the culture period, using a color camera. An oxygen gradient was thus maintained across the intestinal barrier. The hypoxic culture conditions on the gut side of the chip helped maintain an ideal environment for the bacteria. [21]. Reproduced with permission from Nature Biomedical Engineering; published by Springer Nature, 2019. (**B**) The electrical resistance across a physiological barrier can be an indication of how intact the barrier is. To measure this resistance, an electrode is placed on either side of the barrier. A low amplitude AC electric signal is then applied, and a sweep across a frequency range is performed. By measuring the amplitude and phase of the resulting electrical current, one can determine the resistance of the barrier. This process is call impedance spectroscopy [64]. Reproduced with permission from Journal of Laboratory Automation; published by Elsevier, 2015.

Once the structure is formed, one or more cell types are introduced into the chip. There are numerous methods that can be used, alone or in tandem, to accomplish this. Cells or organoids can be incorporated in a gel before its injection into the chip (as demonstrated by Guo et al. [29] (see Figure 2D), Miller et al. [28] (see Figure 2C), Wang et al. [13] (see Figure 1C), and Sobrino et al. [43]) or cells can be allowed to invade from neighboring channels into the gel after it solidifies, or both, (as demonstrated by Nashimoto et al. [16], see Figure 4F). Alternatively, cells can be incubated in the channels until they coat a membrane (as demonstrated by Pemathilaka et al. [11] (see Figure 1A), Jang et al. [44] and by Jalili-Firoozinezhad et al. [21] (see Figure 5A)) or the walls of a channel (as demonstrated by Trietsch et al. [24] (see Figure 2B) and Price et al. [31]). They can even be seeded in gel channels created by a molding process or 3D printing (as demonstrated by Jiménez-Torres et al. [27] (see Figure 2A) and Zhang et al. [22] (see Figure 4B) respectively).

For a comprehensive review of tubular chip models with lumens see Virumbrales-Muñoz et al. [45].

## 4. Advantages of Perfusable OoC Models and Various Implementations of Flow in OoC Models

A key barrier in OoC models is the difficulty in supplying nutrients effectively to larger organoids. When organoids grow beyond a few hundred microns, a necrotic core can start developing [34]. Introducing flow around the organoid, supplies nutrients more effectively to the organoid, while removing metabolites [17]. This increases the viability of the organoids [13,17] and can allow them to grow larger without developing a necrotic core [13]. Increasing the nutrition can also allow for denser cultures, which can increase cellular crosstalk [46]. Care must be taken to properly calibrate the flow rate, since if the media is exchanged too rapidly, it can decrease the levels of endogenous factors in the local microenvironment [46]. Active flow also allows for dynamic control of the environment in the OoC.

Active flow can also be used to allow crosstalk between various cell types, organoids, or chambers in the OoC. Active media circulation is even used to create multi-organ models that evaluate the interaction between the various organs in the body, as demonstrated by Herland et al. [47] (see Figure 4E), Baert et al. [15] (see Figure 7D), Zhang et al. [22] (see Figure 4B), and Lee et al. [17]. Using active flow, controlled gradients of biological factors can also be maintained long term [48] and modified as needed [18]. Integrated sensors can also be incorporated in the chips to monitor biomarkers secreted by the organoids, molecules such as glucose and lactate, oxygen, and PH etc. [49]. Alternatively, the media can be continuously circulated to various external sensors [49]. Biological factors can then be introduced and their level dynamically adjusted accordingly.

The simplest way to implement perfusion is to flow media through channels that are adjacent to the cell culture chambers, but are separated by pillars or a porous membrane, as implemented by Yamanak et al. [14] (see Figure 1D), Wang et al. [13] (see Figure 1C), Komeya et al. [50] (see Figure 7A), and Baert et al. [15] (see Figure 7D). Although the fluid does not directly flow over the cells in the culture, it still provides nutrients, clears metabolites, and circulates the various factors released by the cells. In other models, the fluid directly flows over the cells, for example, through the lumen of a tube or a channel lined with cells (as implemented by Pemathilaka et al. [11] (see Figure 1A), Trietsch et al. [24] (see Figure 2B), and Herland et al. [47] (see Figure 4E)), through the gel that encapsulates the cells acting as interstitial flow (as implemented by Kramer et al. [51] (see Figure 4A) and Wang et al. [52]), or simply over organoids suspended in media (as implemented by Lee et al. [17]). Networks of tubes embedded in the gel can also be used to circulate media throughout the OoC, as implemented by Zhang et al. [22] (see Figure 4B).

Large organoids can be printed with channels in them. This allows media to reach the interior of the organoid, allowing for larger organoids with better viability and without necrosis. Additionally, the structure of an organoid with internal vasculature can be replicated with such methods. An example of this is a liver organoid that was bioprinted to replicate the structure of the liver lobules, by Grix et al. [39] (see Figure 3B). However, media was not actively perfused into the channels, they simply allowed the media to diffuse into the organoid through the printed channels. In future iterations, they plan to introduce active flow.

Chips that replicate vasculature through the self-assembly of VECs often induce the vessels to anastomose to the main synthetic channels [54], as demonstrated by Sobrino et al. [43] (see Figure 4D). In this way, fluid can be perfused throughout the capillary network that is formed. These networks can be used to study cancer and are often incorporated on chips together with cancer cells, as implemented by Sobrino et al. [43] (see Figure 4D). The mechanisms of metastasis and the effectiveness of various drugs can be tested on these chips. Additionally, blood vessels affect the cells and organs around them, and there is significant crosstalk between the vasculature and the nearby organs [54]. Incorporating vasculature near, or even within, an organoid would allow for a more physiologically complete representation of the organoid [34]. There are a few recent models that have even succeeded in inducing the on-chip vasculature to connect with the vasculature embedded in a tumor, as demonstrated by Nashimoto et al. [16] (see Figure 4F). Recently, some chips have incorporated both blood and lymphatic vasculature on a single chip, as demonstrated by Bachmann et al. [55] and Osaki et al. [56]. VECs and the vessels they produce are heterogenous and adapted to specific organs [54]. Some advanced OoC models use organ specific VECs, as in the multi-organ models developed by Herland et al. [47] (see Figure 4E).

The fluid flow can also be used to apply a shear stress on the cells. This is especially important when modeling the environment of cells that are regularly exposed to such shear stresses in vivo, such as VECs in blood vessels [6,57]. For example, the level of shear stress in blood can influence cellular alignment [57] as well as internal cytoskeleton arrangement [58], the creation of new vessels [59], their porosity (barrier function) [31], and the metastasis of cancer cells [6], etc. For models where the cells are grown on a membrane [44] or on the walls of a tube [31], the media is pumped through the channel at a specified rate calculated to exert a specific shear stress on the cells. See Chen et al. [6] for a comprehensive review of the effects of shear stresses induced by various physiological flows in the body and their modeling using microfluidics. Incorporating the appropriate shear stresses, whether peristaltic or steady, in in vitro models helps to induce the natural physiological responses in the cells, improving the accuracy of the models. For example, Huh et al. [60] found that when modeling a gut- in a membrane-style model, peristaltic motion combined with gentle flow induced the spontaneous formation of 3D intestinal villus-like structures (see Figure 5A). Shear stress can also be applied to entire organoids, by inducing flow over the organoids, similar to interstitial flow. For example, Homan et al. [61] found that applying shear stress to kidney organoids enhanced the formation of vascular networks in the organoids. Additionally, the tubular and glomerular compartments in the organoids showed increased maturity. This is in line with the results of a membrane model for the kidney, developed by Jang et al. [44], that included fluid shear stress.

Perfused chips are often used to study the effects of various drugs and toxins on the permeability of various physiological barriers, such as the placental barrier [11] (see Figure 4C), blood–brain barrier (BBB) [62], the intestinal walls [21,24] (see Figure 5A), and blood/lymph vessels [55,63]. Generally, the barrier model is cultured until the cells are confluent. A fluorescent dye of a given molecular weight can then be perfused through the chip, to test the barrier (as demonstrated by Trietsch et al. [24], see Figure 2B). The permeability of the barrier can also be tested by measuring the trans-epithelial electrical resistance (TEER) across the barrier [64] (see Figure 5B, as demonstrated by Goldstein et al. [65] (see Figure 7E)). The permeability of the barrier to a specific drug or molecule can be tested by perfusing it through one channel and analyzing the media in the second channel, across the barrier, for its presence. This can be done, either by analyzing the media off-chip using advanced tools such as mass spectroscopy (as demonstrated by Pemathilaka et al. [11]), or in real time using sensors to monitor the concentration levels in the media on one or both sides of the barrier, such as the oxygen sensors implemented on-chip by Jalili-Firoozinezhad et al. [21] (see Figure 5A).

Perfusion can also be used to mimic interstitial flow, providing some of the physical cues that it provides in vivo [66]. For example, interstitial perfusion flow can help induce, promote, and maintain blood [67] and lymphatic [55] vascularization of the OoC. Kramer et al. [51] (see Figure 4A) found that interstitial flow decreased the proliferation of Pancreatic Ductal Adenocarcinoma (PDAC) spheroids, but increased their chemoresistance. By carefully controlling the pressures used to drive the interstitial flow or those generated by the induced flow, the hydrostatic interstitial fluid pressure (IFP) can also be controlled. The induction of such hydrostatic pressure can also physiologically affect the cells [68]. Additionally, the induction of a pressure differential between the IFP and the pressure in a blood/lymph vessel, or the application of a pressure differential across a cell barrier grown on a membrane, can be used to control and study transmural flow and the pressure-gradient-induced transport of nutrients and biological factors across the membrane or walls of the vessels and barriers [55,63].

## 5. Various Pumping Schemes Used

The flow rate required for perfusion in microfluidic devices varies widely, from nL/min [17] to mL/min [57]. The length of culture time also varies widely and can range from hours [11] to months [50]. The large variability necessitates multiple pumping strategies. Another factor to consider is whether the fluid should be recirculated or if a continuous flow of fresh media is preferred. Lastly, the space in the incubator must be considered, as the size of pump can significantly impact the ability to run multiple parallel experiments simultaneously.

**Figure 6:**
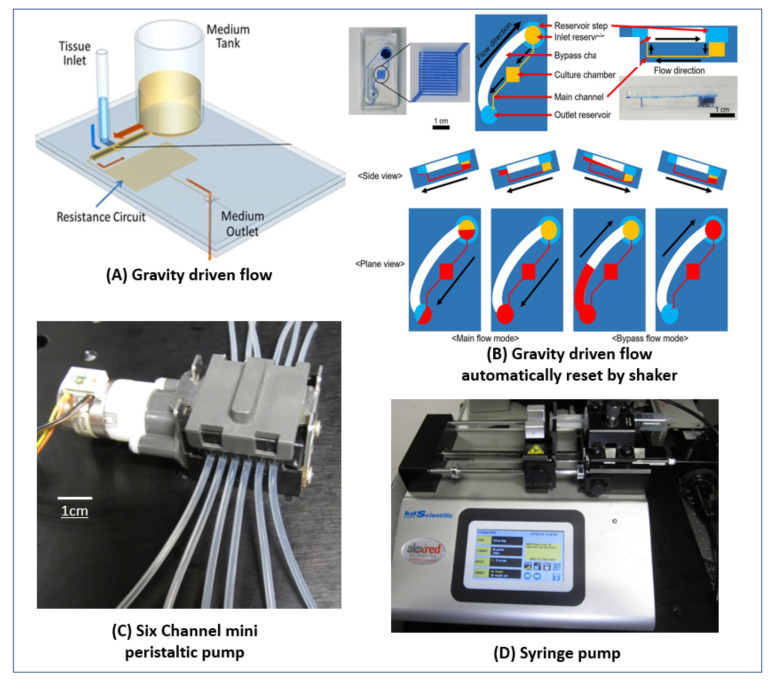
**Pumping strategies:** (**A**) Testicular tissue fragments were cultured under a membrane in the microfluidic channel. A 3-mL reservoir was used to supply fresh medium to the tissue. A long snaking channel was added to introduce hydrodynamic resistance and slow the flow. The reservoir was refilled twice a week [50]. Reproduced with prmission from Scintific Reports; published by Springer Nature, 2017, under a Creative Commons BY 4.0 license (http://creativecommons.org/licenses/by/4.0/ accessed on 1 May 2022). (**B**) A microfluidic chip with flow driven by gravity that can sustain flow indefinitely in a single direction. The chip is kept on a programmable stage that can tilt to various angles at specified intervals. The chip contains two layers of fluid channels. The lower layer contains the inlet resrvoir and the main channel, while the upper layer contains the bypass channel (whose hydrodynamic resistance is much lower than that of the main channel) and the outlet reservoir. In the first stage, the inlet reservoir is filled and the stage is tilted so that medium flows from the inlet reservoir through the main channel and into the outlet reservoir. When most of the media in the inlet reservoir is depleted, the stage tilts in the other direction lowering the inlet reservoir. The media then flows through the low resistance bypass channel and refills the inlet reservoir. In this way, a continuous gravity-powered unidirectional flow can be maintained indefinitely [70]. Reproduced with permisson from Biotechnology Progress; published by Wiley, 2019. (**C**) A mini six-channel peristaltic pump from Takasago Fluidic Systems with a DC stepper motor that can supply flow rates from 0.23 µL/min to 380 µL/min with an accuracy of ±10% between channels. (**D**) A two-channel syringe pump holding a 10-µL glass syringe and a 20-mL plastic syringe. The model is a Legato 210 from KD scientific. With the 10-µL syringe it can supply flow rates as low as 0.067 nL/min, while with the 20-mL syringe it can supply flow rates as high as 54 mL/min. This model can be used with syringes ranging from 0.5-µL to 140 mL.

The simplest method uses gravitational flow. A relatively large and tall reservoir is filled with media, and it gradually flows across the chip to another reservoir with a lower water level. The flow rate can be regulated by controlling the hydrodynamic resistance of the microfluidic channels, as demonstrated by Komeya et al. [50] (see Figure 6A), Price et al. [31], and Sobrino et al. [43]. The main advantages are its simplicity, capability for low flow rates, and that it does not require a source of power nor any external tubing. The drawbacks are that the flow rate is not steady, since as the fluid flows from one reservoir to the second, the height difference decreases, causing the flow rate to decrease as well [69]. Additionally, the fluid does not recirculate, so for longer cultures the fluid level in the reservoir must be reset relatively frequently. Thus, this method is more suitable for slower flow rates or short-term experiments, where the reservoir has sufficient storage capacity to supply enough media for the required duration.

A modification of this method uses a programmable rocker that can tilt and change direction at set intervals, to recirculate the media and maintain a continuous flow, as demonstrated by Trietsch et al. [24], Cho et al. [3], and Kramer et al. [51]). This eliminates the limitation on the duration of gravitational flow, but necessitates a power source and place for the rocker (although one rocker can hold many chips). Additionally, the flow direction changes each time the rocker tilts. A creative modification of this method shown in Figure 6B uses a rocker to reset the device, while still maintaining unidirectional flow, as demonstrated by Lee et al. [70].

Using an electrically controlled pump allows for steady flow rates that can be programmed to change dynamically. Syringe-pumps (see Figure 6D, as used by Pemathilaka et al. [11], Wang et al. [13] Seo et al. [71], Jang et al. [44], and Nashimoto et al. [16]) provide a very steady non-pulsatile flow and have an extremely wide range of flow rates. They can also drive multiple syringes in parallel, for an even flow rate across many chips. However, they cannot recirculate the media [69], limiting their run-time, although some models can pump in both directions and have withdrawal and infusion modes. Generally, they are quite bulky, although some manufacturers have developed small pen-sized syringe pumps, such as the Pen-Type Syringe Pump-SBP Series from Takasago Fluidic Systems.

Peristaltic pumps (as used by Zhang et al. [22], Homan et al. [61] and Hattori et al. [57]) can also handle a very large range of flow rates, from hundreds of nL/min to tens of mL/min, and multi-channel pumps are available (as used by Herland et al. [47] and the small six-channel pump from Takasago Fluidic Systems, see Figure 6C). There are also miniature sized pumps, such as the Ultra-Small Peristaltic Pump from Takasago Fluidic Systems. These pumps can be driven using standard small integrated circuit (IC) driver chips, such as the Easy Driver from Sparkfun. In many designs, the tubing is completely isolated from the pump, and the pump is simply clamped onto the tube; this is an advantage, as it is easier to incorporate and reduces the risk of contamination [69]. In other designs, the pump-head can be replaced or autoclaved for sterility. There are even designs that have incorporated a peristaltic pump into the chip, as demonstrated by Schneider et al. [72] (similar designs are also available commercially, such as the Chip Pump from Takasago Fluidic Systems). The flow can be bidirectional, dynamically controlled, and the media recirculated [69]. The main drawbacks are the somewhat pulsatile flow [69] (depending on the flowrate and the design of the pump, such as the number of rollers) and the generation of bubbles [73] (in our experience, predominantly at very low flow rates). In our experience, the generation of bubbles can be mitigated by increasing the pump outlet pressure. This can be accomplished by introducing a commercial flow resistor, a section of tubing with a very small cross-section, such as (PEEK tubing), or even simply the shaft of a high gauge needle. The bubble generation may also be influenced by the permeability of the tubing used in the pump.

Aside from bubbles generated by a pump, bubbles can also form in any perfusion culture from the evaporation of media through gas-permeable tubing and chips. This can be mitigated by increasing the humidity of the culture chamber or using materials that are less permeable. Inducing flow via infusion of the fluid into the chip rather than withdrawing the fluid can also mitigate bubble formation, since the lower pressure induced by suction can cause air to leach into the chip through the walls of the chip or poor connections.

There are generally two approaches for passively removing bubbles. They can be trapped or they can be removed via a gas permeable material [74]. Simple bubble traps that leverage the buoyancy of air [74] can be implemented in a variety of ways, either on-chip or off-chip. For example, using a vertical fluid column where fluid enters from the top and exits via the bottom with the bubbles rising to the top. This can easily be implemented using a vertical section of a wider diameter tube, in-line with the media supply tube. However, with such methods, the bubbles accumulate and do not dissipate. Eventually, the trap fills, at which point it ceases trapping the bubbles. The second method uses gas permeable materials as debubblers and can handle flow rates as low as 5 µL/min [74]. For example, the fluids can be flowed across a hydrophobic poly(tetrafluoroethylene) (PTFE) membrane, as demonstrated by Williams et al. [74] (similar designs are also commercially available, e.g., the autoclavable “peek bubble remover” from Elveflow). A section of expanded PTFE (ePTFE) tubing in the pump’s outlet line would likely also have a similar effect. These devices require a higher pressure inside the tube relative to the environment, and their performance and maximum flow rate can also be enhanced by applying a vacuum on the other side of the membrane to increase the pressure difference. They are advantageous, as they can run indefinitely. Lee et al. [75] developed a bubble trap that combines elements of both approaches and can run indefinitely without bubble accumulation.

Osmotic pumps can be used for stable flows in the range of hundreds of nL/min and can easily be tuned [69]; for example, the simple pump developed and used by Park et al. [49] and also used by Lee et al. [17]. However, they can only work with hypoosmotic solutions, and a separation needs to be made between the pumped fluid and the media (for example, Park et al. [49] used a small bubble in the tube). Therefore, they can only work in withdrawal mode. They also cannot recirculate the media [69] and need to be reset when the reservoir is emptied; however, at sufficiently low flow rates and with a large enough reservoir this may not be an issue. Electro-osmotic pumps are also suitable for low flow rates and can also be incorporated on-chip. They can be made on-chip by incorporating electrodes, as demonstrated by Glawdel et al. [76], or integrated as an external pump, which are commercially available (such as the Electro-Osmotic Pump-EBP series from Takasago Fluidic Systems). They can be small in size and give a steady non-pulsatile flow. The flow rates however, are sensitive to the conductivity of the medium. High conductivity media, such as that used in cell culture, can cause electrolysis [69]. Some elecro-osmotic pumps only work with specific liquids, such as Deionized Water, Methanol, and Ethanol. Therefore, they are not always practical for cell culture applications or require indirect pumping strategies [69], such as those used with the osmotic pumps discussed above. An advantage over regular osmotic pumps though, is that they are electrically controlled and are bidirectional, allowing for reverse or oscillating flow. Electrothermal pumping can also be implemented on-chip by incorporating electrodes, as demonstrated by Lang et al. [77]. The advantages are that no external tubing or moving parts are required and that any fluid can be used. However, Joule heating can increase the temperature of the culture medium. Care must be taken with the design to ensure that heat is dissipated effectively in order to avoid harming the cells and the various biological factors in the media [77].

Other pumping strategies include, pressure-driven flow, where the inlet and outlet reservoirs are held at different pressures to induce flow, and piezoelectric pumps. Satoh et al. [78] developed a multi-organ pressure-driven platform that drives multiple chips with one pump and can recirculate the media for continuous flow. There are also commercial systems such as the AF1 series microfluidic pump from Elveflow. The flow rate is indirectly determined by the pressure; therefore, care must be taken to ensure all the chips have equal hydrodynamic resistance and are manufactured to close tolerances, since even small changes in resistance, for example different tubing lengths, can impact the flow uniformity across the different chips. Small piezoelectric pumps that are autoclavable are also available, such as the MP6 micropump from Bartels; however, the flow is pulsatile by nature of the actuation mechanism.

For a review of the various pumping techniques that are used in microfluidic cell culture, see Byun et al. [69].

## 6. In Vitro Spermatogenesis

Spermatogenesis is a complex process, whereby spermatogonial stem cells (SSC) divide and differentiate to become haploid round spermatids that eventually undergo spermiogenesis to mature into sperm [79,80]. For many years, scientists have attempted to replicate this process in vitro, with various measures of success. See Huleihel and Lunenfeld [81], Komeya et al. [82], and Richer et al. [83] for comprehensive reviews of the field of in vitro spermatogenesis.

Reproducing the structure and function of the testes in vitro is important for the study of the spermatogenic process and for understanding the cell–cell interactions in the testis and the mechanisms of action of various biological factors that affect testicular function and male fertility. It is also of great clinical importance for pediatric cancer patients facing aggressive gonadotoxic chemotherapy/radiotherapy treatments that can render them sterile. Presently, there are no options available to them to preserve their fertility. Unlike adults, they cannot cryopreserve their sperm as their testes do not yet produce sperm. Current guidelines advise cryopreserving testicular biopsies that contain sperm cell progenitors [84,85,86]. Often however, the tissue cannot be reintroduced, due to the risk of the presence of residual cancer cells that can potentially reintroduce the cancer [84,85,87]. The ability to induce spermatogenesis in vitro and develop fertile sperm would allow them to father their own biological children via in vitro fertilization (IVF), using intracytoplasmic sperm injection (ICSI). In vitro spermatogenesis could also be used in the treatment of azoospermic patients with maturation arrest or even Sertoli cell only syndrome. Their testicular biopsies show no mature sperm, but often they contain sperm progenitor cells at various stages of differentiation, or in some cases only SSCs, that for various reasons are not progressing through spermatogenesis to mature sperm [85,88,89].

There are generally two key approaches to in vitro spermatogenesis. The first method is organ culture, in which a section of testicular tissue is cultured in vitro. This method was shown to induce and maintain spermatogenesis in testicular tissue from mice for a prolonged period of several months. Sato et al. [90] was the first to succeed in generating fertile sperm that was then used to produce live offspring. However, this method is restricted by the size and shape of the original tissue section, thereby limiting the ability to incorporate it into an advanced organ-on-a-chip model. This is especially true for humans where only a limited amount of testicular tissue from the patient is available. The proportions and ratios of the various cell types, the geometry, and, to some extent, the microenvironment are also constrained by the composition of the tissue [82].

The second method utilizes disassociated testicular cells. These cells can be sorted into specific cellular subtypes and cultured, or they can be combined in various proportions and used to form organoids. This method offers greater flexibility; however, although there has been success in replicating some aspects of the in vivo environment, no model has yet generated organoids de novo that completely replicate both the structure and function of the testis and consistently generate mature and fertile sperm [81].

**Figure 7:**
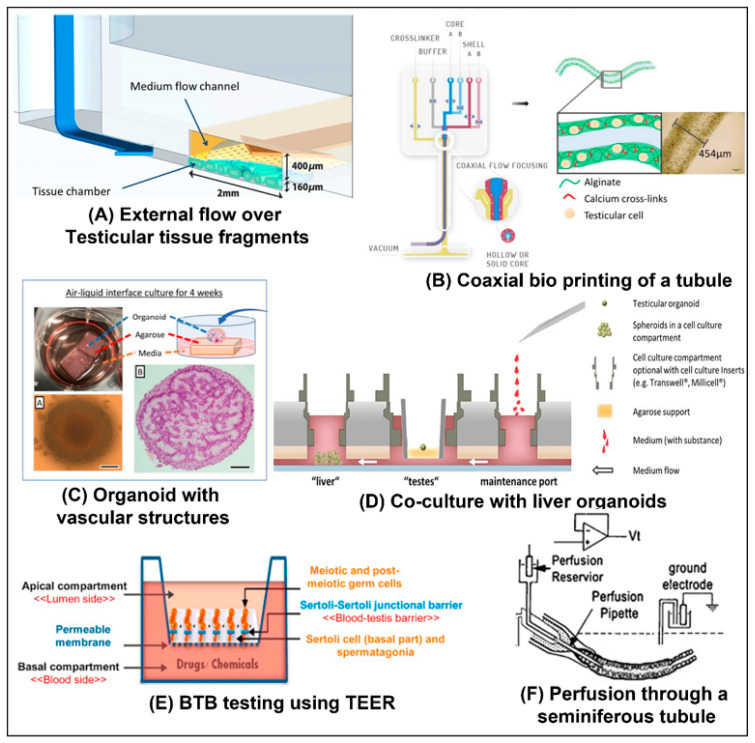
**In vitro spermatogenesis implementations:** (**A**) An organ culture approach was used. Testicular tissue fragments are cultured below a membrane and fresh media is continuously flowed over the membrane. The culture could be maintained for as long as six months [50]. Reproduced with permission from Scintific Reports; published by Springer Nature, 2017, under a Creative Commons BY 4.0 license (http://creativecommons.org/licenses/by/4.0/ accessed on 1 May 2022). (**B**) A seminiferous tubule-like structure was bioprinted. An alginate gel containing a suspension of testicular cells was flowed through a coaxial nozzle, around a sacrificial PVA gel. This resulted in an outer shell of cross-linked alginate that contains the cells and a hollow interior [38]. Reproduced with permission from bioRxiv, 2021, under a Creative Commons BY ND 4.0 license (https://creativecommons.org/licenses/by-nd/4.0/ accessed on 1 May 2022). (**C**) Porcine testicular cells were first cultured in microwells for 24 h, to form organoids. They were then cultured for four weeks on the exposed surface of an agarose base that was partially submerged in media, to provide an air–liquid interface. The organoids developed a structure that included preliminary vasculature [99]. Reproduced with permission from Cells; published by MDPI, 2021. (**D**) Preformed liver organoids and testicular organoids from human cells were cultured in a multi-organ culture chip. The medium was continuously circulated to promote cellular crosstalk [15]. Reproduced with permission from Human Reproduction; published by Oxford University Press, 2020. (**E**) Testicular cells from rats were cultured in Bicameral (two-chamber) well plates. After two days, the Sertoli cells formed a BTB and various toxins were added. The TEER was then monitored to gauge the effect of the toxins on the barrier [65]. Reproduced with permission from Reproductive Toxicology; published by Elsevier 2016. (**F**) A specialized set of three concentric pipettes were used to tightly hold a seminiferous tubule from a rat. A ringer solution was then perfused through the tubule’s lumen, while the trans-epithelial electrical potential was simultaneously measured [102]. Reproduced with permission from Journal of Physiology; published by Wiley, 2002.

Generally, these methods use one of a number of gels, such as Methylcellulose [91], collagen [92], agar [93], Matrigel [94], alginate [95], decellularized testis tissue [96,97], or, in some cases, no gel at all [98]. The organoids are cultured in various conditions, such as a well plate, gel droplets [94], in an air–liquid interface on a block of agar [99] (see Figure 7C), a bioprinted scaffold [95], or, in one study, preformed organoids were cultured in a microfluidic chip [15] (see Figure 7D). Some of these studies have achieved elongated spermatids [91,93,95,100], none yet however, have achieved mature fertile sperm using these methods. Although in one study, using our methylcellulose system, we succeeded in the development of mature sperm, the yield was extremely low and inconsistent, and the fertility of the cells was not proven [93].

Very few of these studies have incorporated microfluidic technologies. This limits the ability to control the microenvironment of the cells, supply nutrition, and remove waste effectively. See Sharma et al. [101] for a comprehensive review of the benefits of utilizing microfluidic technology in replicating in vitro spermatogenesis. This review focuses on incorporating perfusion, active fluid flow, and vasculature into in vitro spermatogenesis models and the significant capabilities it provides beyond simpler microfluidic setups. Additionally, strategies used to recreate tubular structures in vitro can be applied to testicular models, for more spatially- and structurally-accurate replications of the seminiferous tubule structure.

## 7. Current Microfluidic Technologies Used to Model Spermatogenesis In Vitro

Many techniques have been used to replicate spermatogenesis and the testicular structure in vitro (see the above-mentioned reviews, Huleihel and Lunenfeld [81], Komeya et al. [82], and Richer et al. [83]). Described below are a number of the currently-used techniques that are relevant to the implementation of perfusion-based testis-on-chip (ToC) models.

Using an organ culture method, Komeya et al. and Yamanak et al. cultured testicular tissue fragments from pre-pubertal mice in a microfluidic channel under a membrane [50] (See Figure 7A) and behind pillars [14] (see Figure 1D), respectively. Throughout the culture period, media was continuously perfused through the channel. The constant supply of nutrients preserved the viability of the tissue, maintaining spermatogenesis for six months.

Goldstein et al. [65] cultured rat testicular cells on a membrane in a two-chamber device, whose compartments were separated by the membrane (See Figure 7E). The cells then replicated the blood–testis barrier (BTB) de novo and maintained some of the stages of spermatogenesis. They then evaluated the effect of adding four different known testicular toxins: 1,3-dinitrobenzene (DNB), 2-methoxyacetic acid (MAA), bisphenol A (BPA), and lindane. Using an electrode in each chamber, they monitored the TEER (see Figure 5B) to determine how the BTB was affected. Continuously monitoring the resistance showed that some toxins caused transient damage to the barrier, while others caused more permanent damage. Counting the number of meiotic and post-meiotic cells showed the differential effects of the different toxins on the various stages of spermatogenesis, highlighting the difference in their mechanisms of toxicity.

A number of relevant organoid models have also been developed. Cham et al. [99] developed testicular organoids using both fresh and cryopreserved porcine testicular cells (see Figure 7C). After forming organoids in microwells, the organoids were moved to an air–liquid interface setup. Agar blocks were submerged in media with their upper surface exposed. The organoids were placed on top of the blocks and cultured for another four weeks. The organoids’ histology showed both tubular and interstitial compartments. They also developed preliminary vascular structures, which consisted of both immature nascent vessels and vascular structures that were developing into relatively mature micro-vessels, such as capillaries, arterioles, or venules.

A perfused co-culture model was introduced by Baert et. al, who co-cultured liver and testicular organoids on a single OoC chip [15] (see Figure 7D). First the organoids were formed off-chip. They were then transferred to the chip and the effect of cyclophosphamide, a drug to treat cancer that can only harm germ cells after bioactivation in the liver, was tested. Various biological and metabolic markers were monitored in the testicular organoids. The experiments showed that only the co-culture chips were negatively impacted by the drug, while control chips that only contained testicular cells were not affected. Importantly, this effect was only noticeable in cultures with active flow. Under static conditions, there was no noticeable effect.

**Figure 8:**
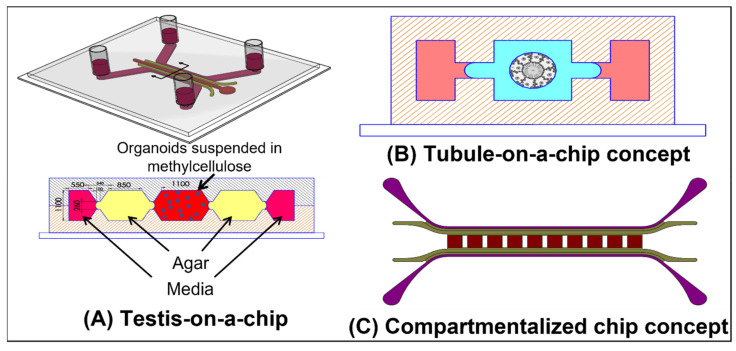
**Testis-on-a-chip concepts from our group:** (**A**) Our testis-on-a-chip design. The chip is made out of PDMS. Disassociated testicular cells are suspended in methylcellulose gel and seeded into the center channel. The next channels (colored in yellow) contain a relatively stiff agar gel, to retain the softer methylcellulose gel. The outermost channels are media channels, for a constant supply of nutrition and clearing of metabolic waste. Shorter capillary barrier-like channels, prevent the spillover of a gel to a neighboring channel during filling [103]. Reproduced with permission from Biofabrication; IOP Publishing, 2022. (**B**) A conceptual model of a tubular model within a gel (blue) flanked by media channels. Image of the tubule cross-section in the center of the model is reproduced with permission from the Journal of Membrane Biology; published by Springer Nature, 2010. (**C**) A conceptual model of separate culture chambers fluidically-connected on the same chip. This would allow co-culture of various organoids and the formation of consistently sized organoids.

Our group, AbuMadighem et al. [103], has recently developed a ToC platform that allows for the development of testicular organoids de novo on-chip (see Figure 8A), as opposed to the above-mentioned models, where the organoids are first formed off-chip. The chip allows for the addition of various biological factors and hormones throughout the culture period. A comparison to traditional static culture in a well-plate showed that there were more meiotic and haploid cells in the culture. Additionally, cell viability was significantly higher. The same chip can also be used for a perfusion culture. A modified design concept containing multiple compartments allows the co-culture of multiple organoid types on a single chip (see Figure 8C). We have found that culturing disassociated cells in a 2.4 mg type 1 rat-tail collagen gel after incubating the channel overnight with a 50 µg collagen solution results in a single organoid in each channel (without the incubation step, multiple organoids are formed). The setup in Figure 8C could thus be used to culture identical organoids, ensuring uniformity in size.

A tubular model was implemented by Robinson et al. [38]. They used a bioprinter with a coaxial printhead to print tubules (see Figure 7B). The tubular shell contained human testicular cells suspended in an alginate bio-ink surrounding a polyvinyl alcohol (PVA) sacrificial core. These tubes were then cultured for 12 days. In some areas a structure containing elements of the seminiferous tubule structure was formed with germ cells in the interior, and Leydig and peritubular cells outside the tube. Analyzing the gene expression of the cells in the tubes showed that functional genes that correspond to the self-renewal of SSCs, meiosis, and spermiogenesis were upregulated.

In another study that is relevant to perfusion models, although it was not an in vitro model, Fisher [102] succeeded in actively perfusing fluid through a seminiferous tubule from a rat, to study the fluid produced by the seminiferous tubules. Ringer medium was perfused through the tubule, while simultaneously the trans-epithelial electrical potential was measured (see Figure 7F).

## 8. Future Application of Perfusion Strategies to In Vitro Spermatogenesis

We propose that OoC approaches that incorporate perfusion can significantly improve the ability to recapitulate spermatogenesis in vitro. Organoids can be formed on the chip, or off-chip using existing methods. They can then be cultured in chips with active flow. This would increase the supply of nutrients and the removal of metabolites. Increasing nutrition improves cell viability and reduces the incidence of necrosis in organoids [3]. It also allows for denser cultures, which could improve cellular crosstalk [46]. On-chip sensors could also be incorporated to allow dynamic control of the culture conditions. Active flow also allows for co-culture with various other cell types and OoC models of other organs, as implemented by Baert et al. [15] (see Figure 7D).

The tubular structure of the seminiferous tubules in vivo makes the testis an ideal organ to replicate using the various tubular OoC models described above. Culturing cells on or in the wall of a perfusable tube would be a more physiologically- and geometrically-accurate representation of the tubules structure. It would also allow access to the lumen (see the concept illustrated in Figure 8B). This would allow for evaluation of the BTB in models with a defined and consistent physiological geometry; similar to the studies performed by Goldstein et al. [65], using the membrane model depicted in Figure 7E.

The ionic composition and the PH of the fluid in the seminiferous tubules differs from the interstitial fluid found outside the tubule. This fluid plays a key role in creating a suitable environment for the sperm to mature [104]. The fluid is secreted by the Sertoli cells, and the environment is regulated and maintained by the BTB and various ion channels (including voltage gated channels) and water channels [104]. However, the exact composition and nature of this fluid has as of yet not been fully characterized It is difficult to study in vivo or in tubule sections due to the difficulty in retrieving the liquid for measurement [104]. If one or more of the various channel ion channels in the Sertoli cells malfunction, this can lead to infertility, indicating the importance of understanding the composition of the tubular fluid. See Rato et al. [104] for a detailed review of the seminiferous tubule fluid (STF). Tubular models, such as the concept depicted in Figure 8B, and membrane models, such as the one implemented by Goldstein et al. [65] (see Figure 7E), can provide access to the luminal compartment and would provide an ideal model to study the fluid. Additionally, if full spermatogenesis were achieved, a tubular or membrane model would allow for the continuous harvesting of mature sperm from the lumen of the tubule, without destroying the model.

There is also an electrical potential difference between the tubule’s lumen and the interstitium [102,105]. The role of this potential has not been extensively studied, but it perhaps plays a role in the regulation of the ionic composition of the tubular fluid. Successfully modeling the BTB in either a tubule model or a membrane chip model that incorporates electrodes, would allow for the study of the electrical potential differences across the barrier, in addition, to the barrier’s permeability and TEER. The electrodes could potentially also be used to artificially induce such an electrical potential in an in vitro model.

Adding active flow to such a tubular model, or to other BTB models, would further increase its impact. Mechanical forces, such as shear stresses due to fluid flow, can greatly impact cellular functions, differentiation, morphology, etc. (see Griffith et al. [4], Kaarj [5] and Moraes et al. [7] for in depth reviews). In the seminiferous tubules, there is pressure gradients and peristaltic-like contractions that create pulsatile fluid flow in the seminiferous tubules [106,107,108]. This flow transports the sperm through the tubules. The contractions are caused by the contraction of the peritubular cells surrounding the tubules and are controlled by a number of mechanisms. Their frequency and intensity vary throughout the tubule, depending on the stages predominantly present in that section. Defects in the genes that affect the contractile proteins in peritubular cells can reduce the contractility of the tubules and cause infertility [109]. The above-mentioned studies focus on various factors that influence the contractions. However, many physiological flows and forces also influence the cells themselves [6,7], and perhaps the motion and flow influence the spermatogenic process itself, similar to the gut, where the contraction and flow is seemingly only to propel the food forward, however, it has been shown, in on-chip model [60] (Figure 5A), that the motion and flow has a significant effect on the morphology and function of the gut model. A perfusion model with either a membrane setup or a tubular model with a lumen would provide an ideal platform to further explore the effects of both the flow and the shear stresses that it applies on the cells, as well as the contractile forces. Recapitulating the BTB in such a model would also allow the study of pressure gradients across the barrier, by maintaining different hydrostatic pressures in the channels on either side of the membrane, or in a tubular model, by maintaining a different pressure in the lumen than in the external chamber, similar to the studies performed by Offeddu et al. [63] and Bachmann et al. [55] with vasculature. Even in organ culture models, flow can be introduced into the tubules using the techniques demonstrated by Fisher [102] (see Figure 7F).

In addition to supplying nutrition, the vasculature interacts significantly with the cells in an organ [54]. In the testis, for example, research has shown that undifferentiated spermatogonial stem cells (SSC) are preferentially localized near the blood vessels in the testes and then migrate when they differentiate [110,111]. Therefore, it would be very significant to culture testicular organoids on vascularized chips [35] perhaps even using testis-specific VECs. This can be implemented by lining the side channels of a chip with VECs, or by incorporating in the gel of the cell culture channel, or a neighboring channel, a hollow tube the lined with VECs. Alternatively, VECs can be induced to self-assemble into vasculature, in the same channel as the organoids. A further extension would entail culturing organoids that already contain elements of vasculature, such as those developed by Cham et al. [99] (see Figure 7C), on a chip that includes vasculature, and inducing anastomosis between the two vasculature networks. This could lead to perfusable and vascularized organoids, as was achieved with tumors by Nashimoto et al. [16] (see Figure 4F). Various strategies to create such perfusable organoids have been discussed By Zhang et al. [34] and Daniel et al. [54].

In conclusion, there are many potential benefits to including active microfluidic flow in in vitro spermatogenesis and ToC models. Improving the current models would deepen the understanding of the complex process of spermatogenesis. Such improvement would also increase the probability of these systems providing a viable option for preserving the fertility of pre-pubertal cancer patients and potentially provide a treatment option for adults with maturation arrest.

## Data Availability

Not applicable.
